# Supplementary material for: Investigating the drivers for antibiotic use and misuse amongst medical undergraduates–perspectives from a Sri Lankan medical school
Source: PLOS Glob Public Health. 2023 Mar 20;3(3):e0001740. doi: 10.1371/journal.pgph.0001740 (PMC10027203; doi:10.1371/journal.pgph.0001740)
Supplement: S1 Text — (DOCX) [file pgph.0001740.s001.docx]

**S1 Text – Questionnaire**

**QUESTIONNAIRE**

Age: ………………………………

Gender: …………………………….

Batch: ………………………………

Is there any family member related to health sector?

If yes, specify:

- - Medical officer
  - Consultant
  - Nursing officer
  - Pharmacist
  - Other

**Knowledge on antibiotics and antibiotic resistance**

Answer the following questions

1. Have you heard the term “Antibiotics”?
   1. Yes
   2. No
2. What would be the definition antibiotics as you understand from the following?
   1. Drug that act against all micro-organisms
   2. Drug that can be used in any illness
   3. Drug that only act against specific bacteria
   4. Drug that only act against specific viruses
   5. I have no idea
3. Do you know what “antibiotic resistance” is?
   1. Yes
   2. No
4. Can you briefly define the above term (you can use your own terms to define)?

………………………………………………………………………………………………………………………………………………………………………………………………………………………………………………………………………………………………………………………………………….

**Answer the following statements by using strongly agree, agree, don’t know, disagree & strongly disagree**

|  | **Strongly agree** | **Agree** | **Don’t know** | **Disagree** | **Strongly disagree** |
| --- | --- | --- | --- | --- | --- |
| 1. Antibiotics are medicine that fight infections caused by bacteria in humans & animals by either killing the bacteria or inhibiting their multiplication |  |  |  |  |  |
| 1. Antibiotic resistance happens when bacterial strains develop ability to withstand the effect of drugs used to treat infections caused by them |  |  |  |  |  |
| 1. Antibiotic resistance means that the human body is becoming resistant to antibiotics |  |  |  |  |  |
| Antibiotics are essential for the following conditions | | | | | |
| - 1. Common cold and flu |  |  |  |  |  |
| - 1. Any Sore throat |  |  |  |  |  |
| - 1. All fevers |  |  |  |  |  |
| - 1. To relieve bodily pain |  |  |  |  |  |
| - 1. Headache |  |  |  |  |  |
| - 1. Urinary tract infections |  |  |  |  |  |
| - 1. Skin and soft tissue infections |  |  |  |  |  |
| - 1. Strep throat |  |  |  |  |  |
| - 1. All cases of vomiting / Diarrhoea |  |  |  |  |  |
| - 1. As prophylaxis for some specific infection |  |  |  |  |  |
| If you agree to the last statement above, Please mention such condition/s (maximum 3 answers) |  | | | | |
| The following actions contribute to emergence of antibiotic resistance | | | | | |
| 1. Using antibiotics without prescription |  |  |  |  |  |
| 1. Taking antibiotics for self-limiting diseases |  |  |  |  |  |
| 1. Over-prescription of antibiotics by healthcare professionals in some clinics |  |  |  |  |  |
| 1. Lack of hygiene and poor sanitation |  |  |  |  |  |
| 1. Poor infection control in hospitals and clinics |  |  |  |  |  |
| 1. Incomplete treatments with antibiotics |  |  |  |  |  |
| 1. Overuse of antibiotics in animals |  |  |  |  |  |
| Antibiotic resistance affect the people as follows | | | | | |
| - 1. Prolonged morbidity |  |  |  |  |  |
| - 1. Prolonged hospitalization |  |  |  |  |  |
| - 1. Risk of mortality |  |  |  |  |  |
| - 1. Increased medical cost |  |  |  |  |  |

**History on antibiotics use**

1. How often do you take antibiotics in a year?
   1. Once
   2. Twice
   3. 2-3 times
   4. More than thrice
   5. Not sure
   6. I didn't take any antibiotics during the last year
2. When did you last take antibiotics?
   1. During last month
   2. During last 3 months
   3. During last 6 months
   4. During the last one year
   5. More than a year ago
   6. Never
3. Have you ever taken antibiotics in the following circumstances?

|  | **Yes, with prescription** | **Yes, without prescription** | **No** |
| --- | --- | --- | --- |
| Cold |  |  |  |
| Sore throat |  |  |  |
| Non-specific fever |  |  |  |
| Headache |  |  |  |
| Wound infection |  |  |  |
| Urinary tract infection |  |  |  |
| Diarrhoea |  |  |  |

If you took antibiotics for other conditions you had recently please mention them here ( joint pains, etc.) ………………………………………………………………………………………………………………………………………………………………………………………………………………………………………………………………………………………………………………………………………………………………………………………………………………………………………………………………………………………………………………………………………………………..

1. For what occasion did you take antibiotics lastly?..................................................................................
2. On that time did you get antibiotics under prescription?
   - 1. Yes
     2. No
3. If yes, how did you get the prescription?
   - 1. After a thorough medical examination
     2. After performing appropriate diagnostic tests recommended by the doctor
     3. After communicating with the doctor via phone
     4. Writing the prescription by myself
     5. Getting prescription from a family member
4. Mark/Tick one of the antibiotics that you have taken during the past 3 months
   1. Amoxicillin
   2. Doxycycline
   3. Cephalexin
   4. Ciprofloxacin
   5. Clindamycin
   6. Metronidazole
   7. Azithromycin
   8. Levofloxacin
   9. Not sure about the name
   10. I haven't taken antibiotics for the past three months
5. How often did you take your antibiotics under indication?
   1. Once a day
   2. 2 times/day
   3. 3 times/day
6. Did you complete the full course of antibiotics prescribed?
   1. Yes
   2. No
7. If yes, how long had you taken antibiotics?
   1. Less than 3 days
   2. Less than 7 days
   3. More than a week
8. Did you recover completely with the antibiotics?
   1. Yes
   2. No
9. If not, what was your next course of action?

|  | Yes | No |
| --- | --- | --- |
| Consulted the same doctor again |  |  |
| I sought another doctor |  |  |
| I took same antibiotics again by my own |  |  |
| I changed to other antibiotics for my own |  |  |

1. Have you ever taken antibiotics without a prescription by a doctor?

a. Yes

b. No

If yes, please answer the following 3 questions:

1. How did you get the antibiotics?
   1. By my own leftover
   2. Bought from a pharmacy
   3. Got them from family members/friends
2. Do you often take antibiotics on your own?
   1. Yes
   2. No
3. Regarding the last time you took antibiotics without a doctor’s prescription, why didn’t you consult a doctor for your illness?
   1. I have some leftovers from last time
   2. I know the drug that doctor would prescribe
   3. I always take some antibiotics for those illnesses
   4. I have leant the drug that would be prescribed
   5. Other (Specify)
4. Have you ever prescribed antibiotics to your friends, family member or to yourself?
   1. Yes
   2. No
5. Do you think that such prescribing as a medical student is acceptable?
   1. Yes
   2. No

If Yes, why do you think so? ............................................................................................................................................................................................................................................................................................................................................

If No, why did you prescribed the antibiotics to them or to yourself? ……………………………………………………………………………………………………………………………………………………………………………………………………………………………………………………………………………………………………………………………………….

1. Have you ever given antibiotics to animals?
   1. Yes
   2. No
2. Have you used left-over antibiotics on yourself or others?
   1. Yes
   2. No
3. Do you generally complete a full course of antibiotics?
   1. Yes
   2. No
4. Do you take antibiotics in the prescribed dosage regime?
   1. Yes
   2. No
5. If you have any leftover antibiotics due to any reason, what do you do to them?
   1. Keep for some time for other occasion
   2. Discard
6. How do you discard any left-over antibiotics?
   1. Disposing medicines with household trash
   2. Flushing medicines in the toilet
   3. Throw to the environment
   4. Other (specify)
7. Would any of these behaviors contribute to antibiotic resistance? State your opinion.

………………………………………………………………………………………………………………………………………………………………………………………………………………………………………………………………………………………………………………………………………

**Attitudes towards antibiotic misuse and antibiotics resistance (ABR) as a health problem**

|  | Strongly agree | Agree | Don’t know | Disagree | Strongly disagree |
| --- | --- | --- | --- | --- | --- |
| - Do you think it is acceptable to take antibiotics without seeing a qualified doctor? |  |  |  |  |  |
| - It is safe to use antibiotics whenever we think they are needed |  |  |  |  |  |
| - I think I have sufficient knowledge on antibiotic use |  |  |  |  |  |
| - Do you agree that antibiotic over prescription contributes to the development of resistance? |  |  |  |  |  |
| - It is essential to give important facts to the patients while prescribing antibiotics in order to prevent antibiotics misuse and over use |  |  |  |  |  |

**Answer the following questions by using agree/disagree**

|  | Agree | Disagree |
| --- | --- | --- |
| - I have taken greater precautions when using antibiotics after learning about them |  |  |
| - My knowledge on Antibiotics and antibiotic resistance is adequate |  |  |
| - I've informed family and friends about antibiotic resistance |  |  |
| - I know when antibiotics are needed |  |  |
| - I suspend antibiotics when the patient feels better |  |  |
| - It is good to prescribe broad spectrum antibiotics at the beginning of antibiotic treatment indiscretely |  |  |
