# Supplementary material for: Investigating the drivers for antibiotic use and misuse amongst medical undergraduates–perspectives from a Sri Lankan medical school
Source: PLOS Glob Public Health. 2023 Mar 20;3(3):e0001740. doi: 10.1371/journal.pgph.0001740 (PMC10027203; doi:10.1371/journal.pgph.0001740)
Supplement: S1 Table — (DOCX) [file pgph.0001740.s002.docx]

**S1 Table. Marking scheme for the questionnaire**

**1. Knowledge score**

*Knowledge score marking scheme*

| **Questions considered** | **Answers** | **Correct/ Incorrect** | **Marks allocated** |
| --- | --- | --- | --- |
| 1. What would be the definition of antibiotics as you understand from the following?  (single answer question) | Drug that act against all micro-organisms | incorrect | 0 |
|  | Drug that can be used in any illness | incorrect | 0 |
|  | Drug that only act against specific bacteria | correct | 1 |
|  | Drug that only act against specific viruses | incorrect | 0 |
|  | (e) (Blank) | incorrect | 0 |
| 2. Can you briefly define the above term (you can use your own terms) | **[stems identified]**  1=Bacteria lose sensitivity, develop resistance against (certain) antibiotics | correct | 1 |
|  | 2=Previously susceptible; now cannot be killed or controlled due to evolution, development of mechanisms, acquiring mutations to develop resistance |  |  |
|  | 3=Bacteria produces mutations |  |  |
|  | 4=Using antibiotics for a long time, wrong dosem misuse, irrational use lead to resistance |  |  |
|  | 5=Body develops resistance against antibiotics | incorrect | 0 |
|  | 6=Other | correct/ incorrect | 1/0 |

| **Answer the following statements by using strongly agree, agree, don’t know, disagree & strongly disagree** | | | | | |
| --- | --- | --- | --- | --- | --- |
|  | **(a) Strongly agree** | **(b)**  **Agree** | **(c)**  **Do not know** | **(d)**  **Disagree** | **(e)**  **Strongly disagree** |
| 3. Antibiotics are medicine that fight infections caused by bacteria in humans & animals by either killing the bacteria or inhibiting their multiplication | 1 | 1 | 0 | 0 | 0 |
| 4. Antibiotic resistance happens when bacterial strains develop ability to withstand the effect of drugs used to treat infections caused by them | 1 | 1 | 0 | 0 | 0 |
| 5. Antibiotic resistance means that the human body is becoming resistant to antibiotics | 0 | 0 | 0 | 1 | 1 |
| **Antibiotics are essential for the following conditions** | | | | | |
|  | **(a) Strongly agree** | **(b)**  **Agree** | **(c)**  **Do not know** | **(d)**  **Disagree** | **(e)**  **Strongly disagree** |
| 6. Common cold & flu | 0 | 0 | 0 | 1 | 1 |
| 7. Any sore throat | 0 | 0 | 0 | 1 | 1 |
| 8. All fevers | 0 | 0 | 0 | 1 | 1 |
| 9. To relieve bodily pain | 0 | 0 | 0 | 1 | 1 |
| 10. Headache | 0 | 0 | 0 | 1 | 1 |
| 11. Strep throat | 1 | 1 | 0 | 0 | 0 |
| 12. All cases of vomiting / Diarrhoea | 0 | 0 | 0 | 1 | 1 |
| 13. As prophylaxis for some specific infection | 1 | 1 | 0 | 0 | 0 |
| **The following actions contribute to emergence of antibiotic resistance** | | | | | |
|  | **(a) Strongly agree** | **(b)**  **Agree** | **(c)**  **Do not know** | **(d)**  **Disagree** | **(e)**  **Strongly disagree** |
| 14. Using antibiotics without prescription | 1 | 1 | 0 | 0 | 0 |
| 15. Taking antibiotics for self-limiting infections | 1 | 1 | 0 | 0 | 0 |
| 16. Over-prescription for antibiotics by healthcare professionals in some clinics | 1 | 1 | 0 | 0 | 0 |
| 17. Poor infection control in hospitals & clinics | 1 | 1 | 0 | 0 | 0 |
| 18. Incomplete treatments with antibiotics | 1 | 1 | 0 | 0 | 0 |
| 19. Overuse of antibiotics in animals | 1 | 1 | 0 | 0 | 0 |
| **Antibiotic resistance affects the people as following** | | | | | |
|  | **(a) Strongly agree** | **(b)**  **Agree** | **(c)**  **Do not know** | **(d)**  **Disagree** | **(e)**  **Strongly disagree** |
| 20. Prolonged morbidity | 1 | 1 | 0 | 0 | 0 |
| 21. Prolonged hospitalization | 1 | 1 | 0 | 0 | 0 |
| 22. Risk of mortality | 1 | 1 | 0 | 0 | 0 |
| 23. Increased medical cost | 1 | 1 | 0 | 0 | 0 |

Knowledge score total = /23 x 100%

**Practice score**

History of antibiotic use and attitudes towards antibiotic misuse were assessed by the second part of self-administered questionnaire and calculated as a practice score.

*Marking scheme for Practice score*

| **Have you ever taken antibiotics in the following circumstances?** | | | |
| --- | --- | --- | --- |
|  | **(a)**  **Yes, with prescription** | **(b)**  **Yes, without prescription** | **(c)**  **No** |
| 1. Cold | 1 | 0 | 1 |
| 2. Sore throat | 1 | 0 | 1 |
| 3. Non-specific fever | 1 | 0 | 1 |
| 4. Headache | 1 | 0 | 1 |
| 5. Wound infection | 1 | 0 | 1 |
| 6. Urinary tract infection | 1 | 0 | 1 |
| 7. Diarrhoea | 1 | 0 | 1 |

|  | **Yes** | **No** |
| --- | --- | --- |
| 8. Have you ever taken antibiotics without a prescription by a doctor? | 0 | 1 |
| 9. Have you ever prescribed antibiotics to your friends, family member or to yourself? | 0 | 1 |
| 10. Have you ever given antibiotics to animal/s? | 0 | 1 |
| 11. Have you used left-over antibiotics on yourself or others? | 0 | 1 |
| 12. Do you generally complete a full course of antibiotics? | 1 | 0 |
| 13. Do you take antibiotics in the prescribed dosage regime? | 1 | 0 |
|  | **Agree** | **Disagree** |
| 14. I have taken greater precautions when using antibiotics after learning about them | 1 | 0 |
| 15. I've informed family and friends about antibiotic resistance | 1 | 0 |

Practice score total = /15 x 100%
