# Supplementary material for: Investigating the drivers for antibiotic use and misuse amongst medical undergraduates–perspectives from a Sri Lankan medical school
Source: PLOS Glob Public Health. 2023 Mar 20;3(3):e0001740. doi: 10.1371/journal.pgph.0001740 (PMC10027203; doi:10.1371/journal.pgph.0001740)
Supplement: S2 Table — (DOCX) [file pgph.0001740.s003.docx]

S2 Table. Factors associated with antibiotic misuse

|  |  | **Total, %**  **(n=347)** | **Gender** | | **Significance** | **Having a family member related to “health sector” at home** | | **Significance** | **Year of study** | | | | **Significance** | **Median knowledge core** | **Significance** |
| --- | --- | --- | --- | --- | --- | --- | --- | --- | --- | --- | --- | --- | --- | --- | --- |
|  |  |  | **Female, %**  **(n=205)** | **Male**  **(n=142)** |  | **No,%**  **(n=239)** | **Yes, %**  **(n=108)** |  | **4^th^ year, %**  **(n=75)** | **3^rd^ year, %**  **(n=76)** | **2^nd^ year %**  **(n=103)** | **1^st^ year, %**  **(n=93)** |  |  |  |
| Ever taken antibiotics without a prescription by a doctor? | No | 186  (53.6%) | 120  (58.5%) | 66 (46.5%) | 0.029* | 128  (53.6%) | 58  (53.7%) | 1.000 | 32  (42.7%) | 46  (60.5%) | 59  (57.3%) | 49  (52.7%) | 0.131 | 91.3 (72.8 - 95.6) | 0.146 |
|  | Yes | 161  (46.4%) | 85  (41.5%) | 76  (53.5%) |  | 111  (46.4%) | 50  (46.3%) |  | 43  (57.3%) | 30  (39.5%) | 44  (42.7%) | 44  (47.3%) |  | 87.0 (69.6 - 95.7) |  |
| Do you often take antibiotics on your own? | (Blank) | 187  (53.9%) | 121  (59.0%) | 66  (46.5%) | 0.062 | 128  (53.6%) | 59  (54.6%) | 0.814 | 32  (42.7%) | 46  (60.5%) | 59  (57.3%) | 50  (53.8%) | 0.361 | 91.3 (69.6-95.7) | 0.335 |
|  | No | 135  (38.9%) | 72  (35.1%) | 63  (44.4%) |  | 95  (39.7%) | 40  (37.0%) |  | 35  (46.7%) | 27  (35.5%) | 37  (35.9%) | 36  (38.7%) |  | 87.0 (73.9 - 95.7) |  |
|  | Yes | 25  (7.2%) | 12  (5.9%) | 13  (9.2%) |  | 16  (6.7%) | 9  (8.3%) |  | 8  (10.7%) | 3  (3.9%) | 7  (6.8%) | 7  (7.5%) |  | 87.0 (58.7 – 93.5) |  |
| Have you ever prescribed antibiotics to your friends, family member or to yourself? | No | 296  (85.3%) | 179  (87.3%) | 117  (82.4%) | 0.220 | 196  (82.0%) | 100  (92.6%) | 0.013* | 56  (74.7%) | 66  (86.8%) | 92  (89.3%) | 82  (88.2%) | 0.031* | 87.0 (69.6 – 95.7) | 0.852 |
|  | Yes | 51  (14.7%) | 26  (12.7%) | 25  (17.6%) |  | 8  (7.4%) | 43  (18.0%) |  | 19  (25.3%) | 10  (13.2%) | 11  (10.7%) | 11  (11.8%) |  | 87.0 (78.3-95.7) |  |
| Do you think that such prescribing as a medical student is acceptable? | No | 329  (94.8%) | 198  (96.6%) | 131  (92.3%) | 0.087* | 226  (94.6%) | 103  (95.4%) | 0.802 | 68  (90.7%) | 72  (94.7%) | 99  (96.1%) | 90  (96.8%) | 0.343 | 87.0 (69.6 – 95.7) | 0.223 |
|  | Yes | 18  (5.2%) | 7  (3.4%) | 11  (7.7%) |  | 13  (5.4%) | 5  (4.6 %) |  | 7  (9.3%) | 4  (5.3%) | 4  (3.9%) | 3  (3.2%) |  | 93.5 (78.3 – 95.7) |  |
| Have you ever given antibiotics to animal/s? | No | 286  (82.4%) | 166  (81.0%) | 120  (84.5%) | 0.474 | 206  (86.2%) | 80  (74.1%) | 0.007* | 61  (81.3%) | 60  (78.9%) | 85  (82.5%) | 80  (86.0%) | 0.688 | 87.0 (69.6 – 95.7) | 0.038** |
|  | Yes | 61  (17.6%) | 39  (19.0%) | 22  (15.5%) |  | 33  (13.8%) | 28  (25.9%) |  | 14  (18.7%) | 16  (21.1%) | 18  (17.5%) | 13  (14.0%) |  | 91.3 (82.6 – 95.7) |  |
| Have you used left-over antibiotics on yourself or others? | No | 273  (78.7%) | 169  (82.4%) | 104  (73.2%) | 0.046* | 186  (77.8%) | 87  (80.6%) | 0.576 | 62  (82.7%) | 56  (73.7%) | 82  (79.6%) | 73  (78.5%) | 0.598 | 87.0 (71.7 – 95.7) | 0.234 |
|  | Yes | 74  (21.3%) | 36  (17.6%) | 38  (26.8%) |  | 53  (22.2%) | 21  (19.4%) |  | 13  (17.3%) | 20  (26.3%) | 21  (20.4%) | 20  (21.5%) |  | 87.0 (68.5 – 91.3) |  |
| Do you generally complete a full course of antibiotics? | No | 81  (23.3%) | 61  (29.8%) | 20  (14.1%) | 0.001* | 55  (23.0%) | 26  (24.1%) | 0.891 | 8  (10.7%) | 20  (26.3%) | 28  (27.2%) | 25  (26.9%) | 0.035* | 82.6 (60.9 – 95.7) | 0.027** |
|  | Yes | 266  (76.7%) | 144  (70.2%) | 122  (85.9%) |  | 184  (77.0%) | 82  (75.9%) |  | 67  (89.3%) | 56  (73.7%) | 75  (72.8%) | 68  (73.1%) |  | 89.1 (73.9 – 95.7) |  |
| Do you take antibiotics in the prescribed dosage regime? | No | 24  (6.9%) | 15  (7.3%) | 9  (6.3%) | 0.831 | 20  (8.4%) | 4  (3.7%) | 0.169 | 0  (0.0%) | 3  (3.9%) | 8  (7.8%) | 13  (14.0%) | 0.003* | 60.9 (48.9 – 84.8) | <0.001** |
|  | Yes | 323  (93.1%) | 190  (92.7%) | 133  (93.7%) |  | 219  (91.6%) | 104  (96.3%) |  | 75  (100.0%) | 73  (96.1%) | 95  (92.2%) | 80  (86.0%) |  | 87.0 (73.9 – 95.7) |  |

* Chi-square test

** Mann-Whitney U test
