# Supplementary material for: Investigating the drivers for antibiotic use and misuse amongst medical undergraduates–perspectives from a Sri Lankan medical school
Source: PLOS Glob Public Health. 2023 Mar 20;3(3):e0001740. doi: 10.1371/journal.pgph.0001740 (PMC10027203; doi:10.1371/journal.pgph.0001740)
Supplement: Attachment — Submitted filename: Rebuttal only.docx [file pgph.0001740.s005.docx]

*We thank the reviewers for the useful comments. The responses are indicated in Italics.*

Journal Requirements:

1. Please change "female” or "male" to "woman” or "man" as appropriate, when used as a noun (see for instance https://apastyle.apa.org/style-grammar-guidelines/bias-free-language/gender).

*Thank you for the comment. Participants identified their gender in an open ended question as male and female. Therefore, we retained the words. Further, in the context the words are used, the words male and female are more appropriate than men and women. However, we did concert some instances to adjectives as appropriate, e.g male participants, female participants.*

2. PLOS Global Public Health does not copy edit accepted manuscripts (https://journals.plos.org/globalpublichealth/s/criteria-for-publication#loc-5). To that effect, please ensure that your submission is free of typos and grammatical errors.

*We have gone through the manuscript again and made several changes to improve the language of the manuscript. Changes can be identified in track-changes.*

3. Please send a completed 'Competing Interests' statement, including any COIs declared by your co-authors. If you have no competing interests to declare, please state "The authors have declared that no competing interests exist". Otherwise please declare all competing interests beginning with the statement "I have read the journal's policy and the authors of this manuscript have the following competing interests:"

*We have changed the title of the section “conflict of interest to competing interests” and changed the text as suggested.*

4. We have noticed that you have a list of Supporting Information legends "S1 Text. Google forms based self administered questionnaire" in your manuscript. However, there are no corresponding files uploaded to the submission. Please upload them as separate files with the item type 'Supporting Information'.

*We have uploaded the questionnaire as supplementary material.*

5. In the online submission form, you indicated that "The database would be made available to any researcher who has a legitimate requirement, through the corresponding author". All PLOS journals now require all data underlying the findings described in their manuscript to be freely available to other researchers, either 1. In a public repository, 2. Within the manuscript itself, or 3. Uploaded as supplementary information.

This policy applies to all data except where public deposition would breach compliance with the protocol approved by your research ethics board. If your data cannot be made publicly available for ethical or legal reasons (e.g., public availability would compromise patient privacy), please explain your reasons by return email and your exemption request will be escalated to the editor for approval. Your exemption request will be handled independently and will not hold up the peer review process, but will need to be resolved should your manuscript be accepted for publication. One of the Editorial team will then be in touch if there are any issues.

*We have uploaded the database as a supplementary material*

Additional Editor Comments (if provided):

This is an interesting work related to the use of antibiotics among medical undergraduates, however, the manuscript needs to be copyedited and it is necessary to improve the structure of the manuscript specially results and discussion.

*Thank you for the comments. We have gone through the manuscript again to improve the manuscript.*

[Note: HTML markup is below. Please do not edit.]

Reviewers' comments:

Reviewer's Responses to Questions

Comments to the Author

1. Does this manuscript meet PLOS Global Public Health’s publication criteria? Is the manuscript technically sound, and do the data support the conclusions? The manuscript must describe methodologically and ethically rigorous research with conclusions that are appropriately drawn based on the data presented.

Reviewer #1: Yes

Reviewer #2: Yes

2. Has the statistical analysis been performed appropriately and rigorously?

Reviewer #1: Yes

Reviewer #2: N/A

3. Have the authors made all data underlying the findings in their manuscript fully available (please refer to the Data Availability Statement at the start of the manuscript PDF file)?

The PLOS Data policy requires authors to make all data underlying the findings described in their manuscript fully available without restriction, with rare exception. The data should be provided as part of the manuscript or its supporting information, or deposited to a public repository. For example, in addition to summary statistics, the data points behind means, medians and variance measures should be available. If there are restrictions on publicly sharing data—e.g. participant privacy or use of data from a third party—those must be specified.

Reviewer #1: No

Reviewer #2: Yes

*Thank you: We have now uploaded the database as a supplementary material*

4. Is the manuscript presented in an intelligible fashion and written in standard English?

PLOS Global Public Health does not copyedit accepted manuscripts, so the language in submitted articles must be clear, correct, and unambiguous. Any typographical or grammatical errors should be corrected at revision, so please note any specific errors here.

Reviewer #1: Yes

Reviewer #2: Yes

*Thank you. We have made further improvements to the text*

5. Review Comments to the Author

Please use the space provided to explain your answers to the questions above. You may also include additional comments for the author, including concerns about dual publication, research ethics, or publication ethics. (Please upload your review as an attachment if it exceeds 20,000 characters)

Reviewer #1: This is an interesting and novel study that looks at how medical students, who are at various stages of their training in infectious diseases, antibiotic biology and use, and clinical training, use or misuse antibiotics. The study addresses their knowledge, which as expected does change as they progress in medical education, and their practice, which does not. This “knowledge-practice” gaps is particularly interesting.

The study is well done but a few factors should be addressed.

• The questionnaire needs to be provided, either in supplemental data or a fully accessible site. That allows others to use the information to see how this “knowledge-practice” gap varies globally.

*Thank you for the comment, the questionnaire has been uploaded*

• The data availability does not appear to meet PLoS guidelines.

*We have now uploaded the anonymized database*

• The Discussion largely just repeats the Results and does not integrate broader social science studies that have examined this gap between what individuals know and what they do. There is a rich scholarly literature on this and the authors are strongly encouraged to incorporate it into the Discussion in lieu of just repeating the Results. Building on the basis of why individuals do this will be fundamental in addressing this “know-do” gap.

*Thank you. We have re-done the discussion incorporating further literature in to the discussion. We have also included suggestions to bridge the “know-do” gap and hope the reviewers would be satisfied with the changes made.*

Reviewer #2: Thank you for this manuscript.

My comments are in the order they appear in the manuscript.

The title: the word misuse is repetitive, consider changing title to 'Investigating the drivers for antibiotic use and misuse amongst medical undergraduates - perspectives from Sri Lankan medical undergraduates”

*Thank you for the suggestion, we have changed the title of the manuscript to “'Investigating the drivers for antibiotic use and misuse amongst medical undergraduates – perspectives from a Sri Lankan medical school”*

Abstract

Needs to be rewritten to clarify a few things. Are you suggesting the medical student cohorts will misuse antibiotics for themselves? Since they cannot yet prescribe.

*Given that medical students are taught the theory of antibiotics and their use, they are prompted to both self-medicate as well as to prescribe to others. We have changed the sentences to “Medical undergraduates are a unique group who are gaining the theoretical knowledge on prescribing antibiotics but are not legally authorized to prescribe. This unique situation may result in self-medication and unauthorized prescription of antibiotics”*

Remove the word 'done' the study was conducted across xyz

*Thank you, changed as suggested*

And again repetition of the word misuse please rewrite as per title.

*Thank you, changed as suggested. The sentence now reads as …..”to identify the patterns and drivers of antibiotic use and misuse among medical undergraduates”*

How the results are presented are not coherent. Add ratios as well as %s. 347 students with a mean age of 24, of whom 142/347 (40.9%) were male.

The rest of results need to be rewritten for clarity. It is not clear how knowledge was scored.

*Thank you. We have followed the suggested format for writing the results. The methods section in the abstract reads “*Two scores; practice score and knowledge score were calculated to compare with the associated factors*.” We have kept the knowledge score and this statement in the abstract. We hope readers will refer to the results section to identify the exact method of calculation.*

Introduction

AMR in brackets in first line.

*Thank you, done.*

An assumption is being made that medical undergraduates gain theoretical knowledge on AMR. Is there any data from Sri Lanka on how much is taught on AMR and prescribing as part of undergraduate curricula?

*Thank you for the comment. There is no data on this. Sri Lanka has only state owned medical faculties. There is a subject bench mark statement for medicine, that is followed by all faculties. All graduates are sitting for a common merit examination at the end of their undergraduate training. This examination is faced by all medical graduates, irrespective of the graduating university. Therefore, all faculties are expected to learn the core-content. The study site teaches AMR in detail to the students, both the technical details of AMR and the wider public health implications of it. Therefore, the authors wish to keep the statements in the introduction as it is.*

*Further, in the limitations section, we have added the following statements “Our study was conducted among medical students of a single university. However, all medical students in Sri Lanka are selected from a competitive Advanced/Level examination, all faculties prepare students for a common examination at exit, and to full-fill a common subject benchmark statement. Therefore, the population is relatively homogenous and are taught similar curricula, enabling generalization.”*

Page 3 line 57 are you referring to Sri Lanka specifically?

*The sentence reads “There is considerable misuse of antibiotics such as over the counter purchase, consumption without prescription, and use of leftover antibiotics, among medical undergraduates worldwide despite the possession of knowledge on antimicrobials and antimicrobial resistance”. We have replaced one of the references.*

Methods

What were the steps to ensure the questionnaire content was comprehensible, non-offensive, unbiased?

*We have re-phrased this paragraph. Now it reads* *“Review and revision of questionnaire was performed so that is was easily comprehensible, non-offensive and unbiased, though expert validation by a consultant microbiologist and a specialist in medical education along with pilot testing on a sample of 10 students”*

Please provide the questionnaire as a supplementary file.

*Questionnaire is provided*

Data are plural hence data were not data was – *Thank you. Corrected*

Was a calculation made for an appropriate sample size and response rate? If so how and if not why not?

*Thank you for the comment. We have not done a sample size calculation, we have identified this as a shortcoming and included in the limitations section. It reads as “We did not calculate a sample size and used convenience sampling for the study, given the circumstances of COVID-19 pandemic. However, the sample size of 347 is sufficiently powered for the descriptive calculations and association calculations”*

How was knowledge assessed?

*Knowledge was assessed through the knowledge score. We have uploaded the questionnaire. Further, the already uploaded supplementary table gives the marking scheme used. Further, table 3 also gives the details used. The methods section includes the following statement “A practice score and a knowledge score were calculated. (S1 Text)”*

What does throwing to the environment mean?

*Thank you. We have changed this to discarding to environment.*

How was significance assessed? E.g. you need to describe in methods. And are your data powered to apply statistical analyses? What hypothesis are you proving or disproving?

*Thank you for the comment. The methods section already reads as “Distribution of knowledge and practice scores were checked with Shapiro-Wilk test of Normalcy. As the data were not normally distributed, the following tests were used for comparisons; categorical variables were compared across groups with Chi-square or Fisher’s exact test, continuous variables were compared across groups with Mann-Whitney U test or Kruskal-Wallis test.”*

*Further we added the following statement “A p value of less than 0.05 was considered as significant”.*

*The analysis done is a basic association calculation. We have not identified or used the terms risk factors, therefore, we assume that the power of the study is not significant. This is an explorative study, where we plan to use the data gathered to develop a hypothesis than to prove or disprove a hypothesis.*

It is not clear to me to what aspect of the data the thematic analysis refers to. This needs a section in methods and justification of thematic analysis to quantitative data. Or is this to the free text data?

*Thank you for the comment. We added the following statement to the methods section* “Open ended questions were analyzed thematically”

This study is not powered for such representation of data.

*Authors are unclear of the reviewer comments. If the reviewer would like to specify the comment, we would be able to address it.*

Thirteen tables for a study including a survey of 347 participants is not really appropriate. I suggest you review how the data are presented and select as a maximum four tables with key results that are meaningful and relate to your objectives. Same goes for all the power calculations.

*Thank you for the comment. We have removed some of the tables. The current table count is 6 and we have combined the last three tables together as a single Supplementary table. If the reviewers and editors would suggest so, the authors are agreeing to move this as Table 7 in to the text as well.*

Discussion

What is a satisfactory response rate? Also no need to repeat results again in discussion. Do not repeat the results in discussion - rather try and interpret what they mean and what implications they have for addressing AMR in context of Sri Lanka. Discussion needs to be rewritten to remove all results and include a meaningful interpretation of the implications. As well as being shortened significantly and remove the sub headings.

*Thanks very much. We have changed the whole discussion section. We hope the updated discussion is satisfactory.*

This manuscript also needs a limitations section.

*Thank you. We have added a limitations section.*
