# Supplementary material for: Investigating the drivers for antibiotic use and misuse amongst medical undergraduates–perspectives from a Sri Lankan medical school
Source: PLOS Glob Public Health. 2023 Mar 20;3(3):e0001740. doi: 10.1371/journal.pgph.0001740 (PMC10027203; doi:10.1371/journal.pgph.0001740)
Supplement: Attachment — Submitted filename: Rebuttal only.docx [file pgph.0001740.s006.docx]

The manuscript has been copy-edited

As no specific comments were not given, a detailed rebuttal was not drafted.
